# Neuronal Degeneration Impairs Rhythms Between Connected Microcircuits

**DOI:** 10.3389/fncom.2020.00018

**Published:** 2020-03-03

**Authors:** Samantha N. Schumm, David Gabrieli, David F. Meaney

**Affiliations:** ^1^Department of Bioengineering, School of Engineering and Applied Sciences, University of Pennsylvania, Philadelphia, PA, United States; ^2^Penn Center for Brain Injury and Repair, Department of Neurosurgery, Perelman School of Medicine, University of Pennsylvania, Philadelphia, PA, United States

**Keywords:** neurodegeneration, microcircuit, network, synchronization, rhythms

## Abstract

Synchronization of neural activity across brain regions is critical to processes that include perception, learning, and memory. After traumatic brain injury (TBI), neuronal degeneration is one possible effect and can alter communication between neural circuits. Consequently, synchronization between neurons may change and can contribute to both lasting changes in functional brain networks and cognitive impairment in patients. However, fundamental principles relating exactly how TBI at the cellular scale affects synchronization of mesoscale circuits are not well understood. In this work, we use computational networks of Izhikevich integrate-and-fire neurons to study synchronized, oscillatory activity between clusters of neurons, which also adapt according to spike-timing-dependent plasticity (STDP). We study how the connections within and between these neuronal clusters change as unidirectional connections form between the two neuronal populations. In turn, we examine how neuronal deletion, intended to mimic the temporary or permanent loss of neurons in the mesoscale circuit, affects these dynamics. We determine synchronization of two neuronal circuits requires very modest connectivity between these populations; approximately 10% of neurons projecting from one circuit to another circuit will result in high synchronization. In addition, we find that synchronization level inversely affects the strength of connection between neuronal microcircuits – moderately synchronized microcircuits develop stronger intercluster connections than do highly synchronized circuits. Finally, we find that highly synchronized circuits are largely protected against the effects of neuronal deletion but may display changes in frequency properties across circuits with targeted neuronal loss. Together, our results suggest that strongly and weakly connected regions differ in their inherent resilience to damage and may serve different roles in a larger network.

## Introduction

Affecting as many as 3.8 million new patients each year (Langlois et al., 2006), traumatic brain injury (TBI) is a leading cause of disability in the U.S. population (Blennow et al., 2016; Pevzner et al., 2016). As such, TBI constitutes a substantial financial burden for both caregivers and healthcare systems (Coronado et al., 2012; Pevzner et al., 2016). Although TBI may occur during high-contact sports or from exposure to explosive military devices (Blennow et al., 2016), TBI is more frequently caused by motor vehicle accidents and falls (Blennow et al., 2016). In addition, TBI commonly affects the elderly, a growing demographic in the United States.

Due to its diverse array of causes, TBI has broad social impact across many demographics and continues to pose a challenge to researchers attempting to develop treatments. Although many recover completely from mild TBI, other patients suffer long-term consequences (Masel and DeWitt, 2010; Blennow et al., 2016; Hiploylee et al., 2017; Wilson et al., 2017), which include memory deficits, sleep disturbances, or mood disorders (Masel and DeWitt, 2010; Wilson et al., 2017). Recent work shows that some of these long-term effects are associated with lasting changes in brain networks. For instance, increased activation in the default mode network is linked to sustained attention deficits after TBI (Bonnelle et al., 2011). Additionally, alterations in functional brain connectivity are thought to explain motor impairments after mild TBI (Kasahara et al., 2010), can target regions involved in cognitive function (Stevens et al., 2012) and sensory processing (Sours et al., 2015), and can differentially target areas associated with episodic memory (Yan et al., 2016). With the well-known heterogeneity of injury patterns and TBI mechanisms, though, it is difficult to draw direct and consistent associations between an impact, the resulting network changes, and the corresponding behavioral impairments. One critically understudied area is how damage in TBI affects the coordination of circuits at the mesoscale level, where hundreds to thousands of neurons coordinate their relative activation pattern with other areas of the brain, leading to the periodic synchronization of areas throughout the brain during task execution, recall, and learning.

Coherence is an important concept across scales in neural communication and brain networks. When the brain is engaged in a task, anatomical regions exhibiting synchronous activity are believed to participate in executing that task (Logothetis and Wandell, 2004; Damoiseaux et al., 2006; Jilka et al., 2014). Most commonly, temporal correlations in hemodynamic fluctuations (functional MRI BOLD data) are used to determine networks of functionally connected brain regions (Fransson, 2006; Greicius et al., 2009). Beyond defining intrinsic brain networks, synchronization is important at the cellular scale for facilitating communication, as it temporarily binds neurons together into functional ensembles (Bastos et al., 2015; Bocchio et al., 2017). Likewise, learning and memory largely depend on coherence, which enables long-distance communication between brain regions (Dü Zel et al., 2010; Wang et al., 2010). Several human imaging studies demonstrate that TBI disrupts synchronization (Sharp et al., 2011; Venkatesan et al., 2015; Wang et al., 2017), leading to the likely increase or decrease in functional network connectivity that contributes to long-term cognitive effects.

Synchronization has been studied extensively at the whole brain scale, but it has also proved important in microscale neuronal networks (Eytan and Marom, 2006; Penn et al., 2016). Despite our understanding and visualization of whole brain activity, little is known about the way in which smaller scale dynamics give rise to high-level coherence. Although it is expected that cellular dysfunction at the beginning and over the course of neurological disorders will impact the coherence of neural activity throughout the brain, there is remarkably little known about how the structure of a network at the cellular scale can lead to coherence changes at the microcircuit level. Furthermore, macroscale synchronization may obscure greater dynamic variability at a smaller spatial scale. Few computational models have emphasized connections between physically separated neuronal clusters or the flow of information between them (Vicente et al., 2008), so there are many unanswered questions regarding how synchronization emerges in mesoscale circuits and how resilient that behavior is to damage.

In this report, we examine how disrupting an intermediate level of neural computation informs and affects the interpretation of large-scale synchrony. We use a computational model of a neuronal network to make precise manipulations that would not be possible experimentally, with the goal of uncovering the principles of mesoscale synchronization that occur when coupled neuronal networks are traumatically injured. There are few existing studies that examine coherence at this scale (Vicente et al., 2008; Gollo et al., 2014), and we are not aware of any similar efforts to examine the unique intersection between traumatic injury and coherence at the mesoscale. We find that our modeled networks synchronize easily despite relatively modest connections between two microcircuits. Upon simulating the effects of neuronal inactivation or degeneration, we find the simplest model of two connected neuronal populations – i.e. the directed projection of neuronal outputs from one cluster to another – reveals inherent advantages of two levels of interconnectivity between microcircuits. Broadly speaking, our results show that highly interconnected clusters are resilient and highly reliable and moderately interconnected clusters are less resilient and more flexible.

## Materials and Methods

Networks were constructed by assembling and connecting clusters consisting of 1000 neurons each. Two of these clusters were then connected. We prescribed the properties of each cluster independently before connecting the two together.

### Properties of a Single Microcircuit

Each individual cluster consisted of 1000 neurons, 80% of which were excitatory and 20% of which were inhibitory, according to empirical evaluation of cortical tissue (Soriano et al., 2008). To create a network, neurons were represented as nodes placed randomly on the surface of a unit sphere, which eliminated the potential boundary effects of a planar geometry. Synaptic connections were represented as directed edges and added at random according to distributions of excitatory and inhibitory connections experimentally derived by Soriano et al. (2008). Neurons averaged 100 outputs and an average of 80 excitatory and 20 inhibitory inputs.

In networks with spike-timing-dependent plasticity (STDP), edge weights are known to follow a bimodal distribution with most connections pushed toward the lowest and highest possible strengths (Song et al., 2000). Accordingly, the initial synaptic strength of each connection was assigned from a bimodal distribution where networks with greater excitatory strength had a higher proportion of strong, high-weight connections. This distribution was scaled from a minimum strength of 0 to a maximum strength of 4 (peak mV/ms). Inhibitory neurons instead followed a Gaussian distribution of strength with 10% variance ranging from −14 to 0 (peak mV/ms). These ranges were selected such that post-synaptic potentials fell within the range of voltages observed empirically for cortical neurons (Ferster and Jagadeesh, 1992). Conduction delays between neurons were proportional to the distance between two neurons and ranged from 1–8 milliseconds (ms), as derived from experimental work by Swadlow (1985).

### Connecting Multiple Neuronal Microcircuits

**FIGURE 1:**
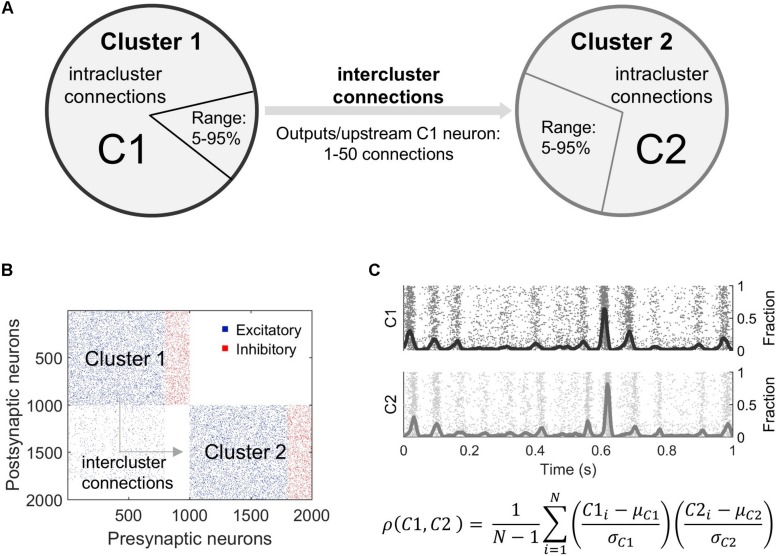
Overview of modeling microcircuit synchronization. **(A)** Two microcircuits (Cluster 1, Cluster 2), each composed of 800 excitatory and 200 inhibitory neurons, were coupled by connecting some outputs of randomly selected neurons in upstream Cluster 1 to randomly selected neurons in downstream Cluster 2. These projections are termed intercluster connections. All neurons were also connected to other neurons within the same cluster via intracluster connections. The relative fraction of neurons in Cluster 1 that sent outputs to Cluster 2 varied from 5 to 95% of the excitatory neuron population in Cluster 1. Similarly, a fraction of excitatory neurons in Cluster 2 was targeted by these outputs (5–95% of excitatory neurons in Cluster 2). The number of intercluster connections from each projecting neuron in Cluster 1 ranged from 1 to 50 downstream connections. **(B)** A connectivity matrix of the overall network topology shows intercluster connections between excitatory neurons in the bottom left quadrant. To mimic *in vivo* connectivity patterns over long distances, only excitatory neurons projected outputs from Cluster 1 to excitatory neurons in Cluster 2. **(C)** Neurons were modeled using the Izhikevich integrate-and-fire formulation. Each simulation achieved a stable firing pattern before activity was analyzed. Raw neuron activity (raster plot) was summed into an aggregate activity trace (solid, oscillating lines) for each cluster and smoothed. Synchronization between the two clusters was calculated as a time-based correlation for 5 min of data. In this equation, ρ is correlation, C1 is Cluster 1, C2 is Cluster 2, μ is the mean, σ is the standard deviation, and *N* is the sample size or number of timesteps.

For more complex simulations, the individual microcircuits (clusters) were first created and then connections were added between them (Figures 1A,B). The parameters defining intercluster connections include the following: (1) the percentage of excitatory neurons in the upstream cluster (“Pre”) that project to the downstream cluster (“Post”), (2) the percentage of excitatory neurons in the downstream cluster that receive connections from the upstream cluster, and (3) the number of connections per upstream projecting neuron (Figure 1A). We randomly selected neurons in the upstream network to connect to randomly selected neurons in the downstream cluster. The synaptic weights for these connections were selected from the weight distribution of the upstream cluster. Intercluster conduction delays were chosen from a uniform distribution in the range of 10 ± 2 ms. This delay corresponds to a separation distance between the two clusters of 2–3 mm. Finally, we maintained the total number of inputs on each excitatory neuron by removing intracluster connections to verify activity-related results are due to the two-cluster architecture and not to a change in the number of inputs a neuron receives. This approach of preserving the number of inputs to a neuron is referred to as “input-degree control.” In a subset of simulations, we compared our results in non-degree controlled and output-degree controlled networks, finding no significant differences between their baseline synchronization behavior. In order to best interpret changes in activity and avoid an unrealistic number of connections, we proceeded with input-degree controlled simulations. Accordingly, we limited the potential number of intercluster connections such that the downstream neurons must receive >50% excitatory inputs from the downstream population, ensuring the downstream cluster remains distinct from the upstream.

To characterize the structural changes with more detail, we identified six subpopulations within the two-cluster topology. There is a total of four excitatory neuron populations defined based on cluster membership (Cluster 1 = presynaptic OR Cluster 2 = postsynaptic) and whether the neurons have intercluster connections. Neurons sending *inter*cluster connections in the upstream or *pre*synaptic cluster are referred to as the *Inter Pre* subpopulation. Neurons with *intra*cluster connections only in the *pre*synaptic cluster are the *Intra Pre* subpopulation. Those receiving *inter*cluster connections in the downstream or *post*synaptic cluster are the *Inter Post* neurons. Finally, neurons with *intra*cluster connections only in the downstream cluster are the *Intra Post* subgroup. There are also two inhibitory neuron populations, one per cluster. These are referred to as *Inhib Pre* and *Inhib Post*. We focused our analysis predominantly on the excitatory subpopulations because these are the neurons that may have intercluster connections and, thereby, shape synchronization most directly (see the section “Results”).

### Dynamics and Neural Activity

Neuron activity was modeled via a system of differential equations, which describe the membrane potential and the recovery potential (Izhikevich, 2003; Izhikevich et al., 2004; Izhikevich and Edelman, 2008; Wiles et al., 2017; Gabrieli et al., 2019). The dynamic equations are as follows:

$$v^{\prime }=0.04\,v^{2}+5\,v+140-u+I$$

$$u^{\prime }=a\,\left(b\,v-u\right)$$

$$\text{if}\,v\geq 30\,\mathrm{mV},\mathrm{then}\,\left\{ \begin{array}{c} v=c\\ u=u+d \end{array}\right.$$

where *v* is the membrane potential in millivolts and *u* is the recovery variable. *I* is the current and includes both synaptically driven and noise currents. The parameters *a*, *b*, *c*, and *d* shape the neuron spiking behavior. These parameters were used to create regular-spiking excitatory neurons and fast-spiking, low-threshold inhibitory neurons, according to Izhikevich (2003).

The model also incorporated primary ionic currents through AMPA and GABA receptors, which drove synaptic-based activity. As in our previous work (Gabrieli et al., 2019), the networks were driven with a contribution of 1 Hz noise according to a gamma distribution (*k*, θ = 2, 1/2) (Izhikevich and Edelman, 2008; Wiles et al., 2017). When neurons fired, the action potential propagated along synaptic connections with a delay depending on the distance the signal must travel. Neurons were desensitized to repeated action potential inputs at 40% attenuation (τ = 150 ms).

Our model also featured STDP in connections between excitatory neurons, according to the following equation:

$$\Delta \,w\,\left(w\right)=\left\{ \begin{array}{c} A_{+}\,\left(w\right)\,\exp\left(-\frac{t_{\text{post}}-t_{\text{pre}}}{\tau }\right)\,\mathrm{if}\,t_{\text{post}}-t_{\text{pre}}>0\\ A_{-}\,\left(w\right)\,\exp\left(-\frac{t_{\text{post}}-t_{\text{pre}}}{\tau }\right)\,\mathrm{if}\,t_{\text{post}}-t_{\text{pre}}\leq 0 \end{array}\right.$$

where *w* is the weight of the connection between two neurons. *A*_+_ and *A*_–_ set the maximum magnitude of synaptic change. *τ* is the plasticity time constant and equal to 20 ms. Finally, *t*_*pre*_ and *t*_*post*_ are the timing of pre- and post-synaptic spikes. By the process of STDP, synapses are strengthened when the post-synaptic neuron fires closely after receiving an input from the presynaptic neuron (Song et al., 2000; Effenberger et al., 2015). If, instead, the post-synaptic neuron fires before receiving a signal from the presynaptic neuron, the synapse is weakened (Song et al., 2000; Effenberger et al., 2015). This process is believed to contribute to learning and memory and to enable entrainment of information into neuronal networks (Song et al., 2000).

Convergence studies were performed by conducting a 24-h simulation and measuring the aggregate change in connectivity weights at each minute over the 24 h of simulation time. The network connectivity reached stable convergence after 90 min. Therefore, we ran all simulations for 2 h to allow adequate time for network activity and synaptic weights to stabilize. All activity and network measures were collected in the final 5 min of simulation time.

To determine synaptic strength parameters used in subsequent simulations, we tested all combinations of excitatory and inhibitory strength available with our model. Given the range of firing rates observed, we then selected one set of strength parameters each for approximately 4, 5, and 6 Hz (Supplementary Figure S1).

### Analysis Metrics

Indeed, there are many ways to measure neural synchronization, ranging from phase locking to different forms of correlation, depending on the relevant time and spatial scales (Varela et al., 2001; Narayanan and Laubach, 2009; Cohen and Kohn, 2011). Here, synchronization of activity between the upstream and downstream clusters was evaluated as a time-based correlation because this methodology incorporated both activity timing and magnitude and was effective for our purposes. That is, we sought to precisely measure the extent to which the population-wide spike density of the downstream cluster matched that of the upstream cluster across minutes of simulated activity. To do so, spiking activity was summed for all neurons of each cluster every millisecond and smoothed with a 50 ms window averaging filter. A filter size of 50 ms was used because it corresponds to an intermediate temporal range of neural activity. This yielded an aggregate, smoothed signal for each cluster (Figure 1C). A time-based correlation was then computed between these two signals and used as a proxy for synchronization.

The rhythmic oscillations of network activity were analyzed with a similar aggregate signal approach. Spike counts were collected in 1 ms bins for the full network, and the resulting signal was then smoothed using a moving average filter (10 ms window) to produce a measure of temporal change in the network spiking activity. The magnitude (height) of the high activity periods (peak prominence ≥ 1) was calculated to represent the relative activation level of the network. The height of each activity peak was normalized by the number of neurons to yield a fraction, and these magnitudes were averaged to obtain a single value for each simulation. In addition, this smoothed, aggregate signal was analyzed in the frequency domain using Welch’s method to generate the power spectral density. The power ratio was computed as the ratio of power in a high frequency band (10–17 Hz) over the power in a low frequency band (1–4 Hz). To identify these bands, we found the highest two peaks in the frequency spectra for all networks considered and determined the range for these two dominant peaks across all spectra. (See Supplementary Figure S2 for more detail and representative spectra for baseline networks).

We used network control theory to identify potentially important roles for subgroups of neurons in the network. Network control theory uses the concept of controllability to identify control points in a network for driving the network to alternative activity states. For example, in the brain, this could mean switching between states of daydreaming and active learning (Gu et al., 2015). Two mechanisms of control are average and modal. Nodes with high average controllability are predicted to be important for driving the network to nearby, easy-to-reach states (Gu et al., 2015). In contrast, nodes with high modal controllability are predicted to drive the network to difficult-to-reach states (Gu et al., 2015). (See Supplementary Figure S3 for schematics). Since the metric relies on the underlying network connectivity to theoretically predict functional roles of nodes, controllability attempts to unite both network structure and function. Using established methods [see Wiles et al. (2017) for derivations] (Gu et al., 2015; Wiles et al., 2017), we calculated both average and modal controllability for each neuron in the network. The raw controllability values were then rank ordered such that 1 is the neuron with lowest controllability and *N* is the neuron with highest controllability.

### Injury

**FIGURE 2:**
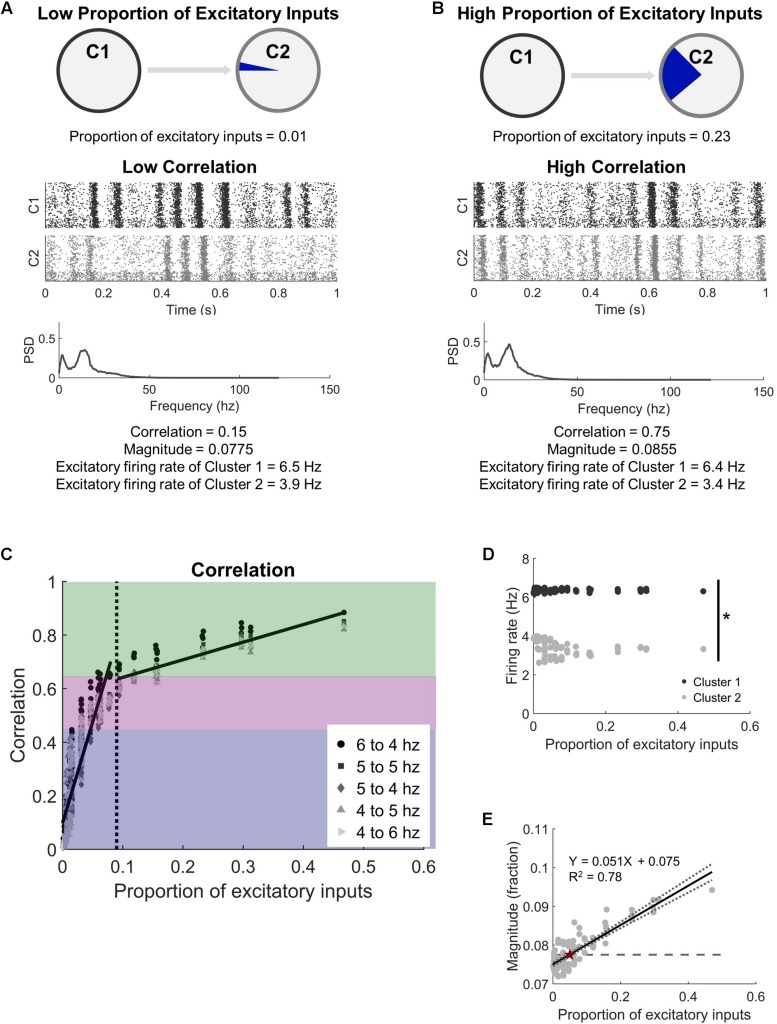
Two microcircuits synchronize activity with relatively few intercluster connections. **(A)** Microcircuits were modeled as two distinct populations of neurons. They were coupled by progressively increasing the proportion of excitatory inputs received by Cluster 2 (C2) from the upstream Cluster 1 (C1). A low proportion of excitatory inputs was associated with low activity correlation between the microcircuits. A representative raster plot of neural activity in both microcircuits shows that periods of high and low activity were not coordinated across the two circuits at low correlation (correlation < 0.45). The corresponding frequency spectrum for low correlation networks has two distinct peaks (PSD = power spectral density). **(B)** In comparison, periods of high and low activity frequently occurred at the same time when the circuits were highly correlated (correlation > 0.65). **(C)** Increasing the proportion of inputs to one microcircuit (C2) from another (C1) led to a rapid increase in synchronization. We considered three regions of synchrony: low (correlation < 0.45; blue region), moderate (0.45 < correlation < 0.65; purple region), and high (correlation > 0.65; green region). Legend indicates the average firing rates of neurons in each microcircuit when correlation is computed. The corresponding frequency spectrum for high correlation networks has two distinct peaks (PSD = power spectral density). **(D)** While the correlation between the two clusters increased with more intercluster connections, the two clusters maintained independent firing rates (*t*-test; *p* < 10^–5^). While the correlation between the two microcircuits increased with more physical connections between them, average firing rates of neurons in each microcircuit were significantly different from each other (Student’s *t*-test; *p* < 10^–5^). **(E)** The magnitude (fraction of network participating) of the high activity oscillations continued to increase with more intercluster connections, showing a strong positive correlation (linear regression, *R*^2^ = 0.78, *p* < 10^–5^). The dashed line marks the baseline level of the null model, which has no intercluster connections. The intersection between the baseline and the regression line is marked with a red star.

To assess the impact of injury on the synchronization of these two neuronal populations, we selected a generic high correlation and moderate correlation network for further analysis. These networks were determined by analyzing the effect of adding intercluster connections in healthy networks. (See the section “Results” and Figure 2 for how these correlation levels were determined). After each network ran for 2 h of simulation time to achieve stable synchronization levels, neurodegeneration was simulated by removing neurons and all their connections from the network. We focused on deleting neurons with a specific structural subtype (see the section “Materials and Methods” for detailed definitions), such that neurons were targeted from a single subtype for each injury simulation. With our interest in testing whether the controllability of a specific neuron was important to overall network synchronization, we first deleted neurons with the highest controllability ranking. For comparison, we deleted the same number of neurons randomly, again by subtype, in separate simulations and compared these results to the targeted deletion approach. After neurons were removed, we ran the simulation for another 2 h to stabilize connectivity weights before analyzing neural dynamics in the final 5 min of the simulation period. This process was repeated for five high correlation and five moderate correlation networks.

### Statistical Analysis

One-way ANOVA was applied to compare the average strength of structural subtypes. A repeated measures model was used to differentiate neuron subpopulations based on nodal network measures. The Tukey–Kramer test was applied *post hoc* for multiple comparisons where relevant. To determine the effects of injuring different neuronal subtypes, we used paired Student’s *t*-test to compare to uninjured baseline measures. Bonferroni corrections were used to determine significance when noted. To compare different neuron selection methods of injury, we applied analysis of covariance (ANCOVA) to control for the injury level covariate.

## Results

### Unidirectional Connection of Two Neuronal Clusters

With our interest in studying how two independent neural circuits synchronize and change after injury, we first studied the physical connectivity requirements for two neural circuits to synchronize their activity. We added unidirectional connections from an upstream Cluster 1 to downstream Cluster 2 (Figures 1A,B) to understand the impact of intercluster connections on network dynamics, namely synchronization (Figure 1C). In general, we observed two phases: (1) a rapidly increasing linear phase of increasing synchronization at low levels of intercluster connection and (2) a more gradually increasing plateau phase at high levels of intercluster connection. We tested different combinations of basal firing rates in Cluster 1 and Cluster 2 and found that these results held for all conditions (Figure 2C). Furthermore, we examined a subset of simulations with 30% inhibitory and 70% excitatory neurons and, again, found this consistent synchronization behavior (Supplementary Figure S4). Importantly, the activity correlation was significantly related to the number of connections between the two clusters. We normalized this quantity as the proportion of excitatory inputs to downstream Cluster 2 that originated in upstream Cluster 1. Using the completely decoupled state of the circuits as the starting point (proportion of excitatory inputs = 0), we found the synchronization level below a proportion of inputs of 0.09 increased rapidly with more intercluster connections (linear regression, *Y* = 7.53*X* + 0.10, *R*^2^ = 0.78, *p* < 10^–5^). Above a physical coupling level of 0.09, the synchronization levels were also significantly correlated with the proportion of intercluster inputs, however more gradually (linear regression, *Y* = 0.66*X* + 0.58, *R*^2^ = 0.77, *p* < 10^–5^). The transition between these two phases occurred around 9% of inputs and was found by determining a cutoff that would produce approximately equal goodness of fit (*R*^2^-values) for both phases. The intersection between the transition point (0.09) and the gradual phase regression line was used to set a threshold for identifying high correlation networks (correlation > 0.65). We also created a designation between moderate and low correlation networks to facilitate subsequent injury analysis where we were interested in networks that could display an appreciable decrease in synchronization. Lastly, we observed relatively modest coupling was required to cause a significant change in synchronization, learning that the downstream cluster needed only 0.3% of inputs from the upstream cluster to significantly change synchronization from baseline (Control networks with 0 intercluster connections: correlation = −0.008 ± 0.006 vs. Networks with 0.3% connection: correlation = 0.025 ± 0.010; paired Student’s *t*-test, *p* = 0.002). For thoroughness, we investigated correlated activity within the excitatory populations of each cluster with similar methodology, finding that Cluster 1 populations (InterPre and IntraPre) are correlated at 0.98 ± 0.01 and Cluster 2 populations (InterPost and IntraPost) are correlated at 0.86 ± 0.09. This result verifies that the two follower populations in the downstream cluster remain coordinated with one another despite our removal of some intracluster connections due to input-degree control.

In addition to synchronization, another important feature of activity in the neural circuits was the rhythmic oscillations of high and low activity that would appear under normal conditions. We converted the signal to the frequency spectrum (Figures 2A,B) to characterize these rhythms and found oscillations of 12.6 ± 0.5 Hz in our uninjured networks. These rhythms are also addressed more formally in Supplementary Figure S2. Unlike synchronization, which plateaued above a specific proportion of intercluster connections, we observed that the rhythmic oscillations continued to include more neurons (higher magnitude) as the coupling of the networks increased (Figure 2E). Peak magnitude showed a strong positive correlation with the proportion of excitatory inputs into Cluster 2 that originate in Cluster 1 (linear regression, *Y* = 0.051*X* + 0.075, *R*^2^ = 0.78, *p* < 10^–5^). We tested whether these changes in synchronous, rhythmic activity were correlated with altered firing rates; however, we found no corresponding change in the average firing rates of the excitatory neurons in the network (Cluster 1: 6.4 ± 0.1 Hz vs. Cluster 2: 3.5 ± 0.4 Hz; paired Student’s *t*-test, *p* < 10^–5^) (Figure 2D). Therefore, the observed increase in correlation depended on a temporal shift in activity in Cluster 2, not increased activity.

**FIGURE 3:**
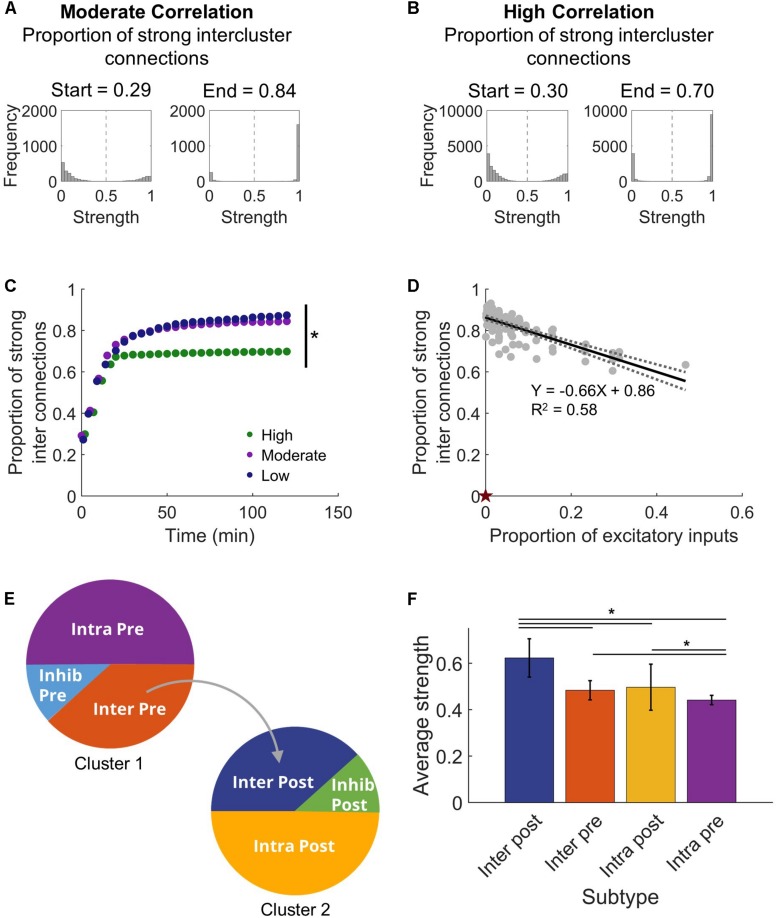
Intercluster connections become strong connections. **(A)** Within either microcircuit, synaptic strength showed a bimodal distribution that is consistent with previous simulations that incorporate plasticity-based changes in synaptic strength. Once connections were made between the circuits, intercluster connections from Cluster 1 to Cluster 2 predominantly increased in synaptic strength. The proportion of strong intercluster connections is defined as the fraction of intercluster connections that have strength greater than half the maximum strength (marked by dashed vertical line in histograms). For a moderate correlation network, the proportion of strong connections was higher at the end time that at the start time. **(B)** Similarly, for a high correlation network, the proportion of strong connections increased from the start time to the end time. **(C)** The proportion of strong intercluster connections (strength > half maximum) increased as the simulation settled, and this proportion remained stable over time for all connected networks. In this representative example, the final proportion was significantly higher in low and moderate correlation networks than in high correlation networks (ANOVA with Tukey’s *post hoc* comparison, *p* < 0.001). **(D)** The proportion of strong synaptic connections between microcircuits depended on the proportion of excitatory inputs. As the number of intercluster connections increased, the proportion of strong intercluster connections decreased (linear regression, *R*^2^ = 0.58, *p* < 10^–5^). The null model has 0 intercluster connections and, thereby, 0 strong intercluster connections (marked by red star). **(E)** We define four excitatory neuron subtypes in this architecture based on their participation in intercluster connections and two inhibitory neuron subtypes. **(F)** The Inter Post neurons had higher average output strength than the other excitatory subtypes (ANOVA with Tukey’s *post hoc* comparison, *p* < 10^–4^).

With this clear change in synchronization that appeared as the network adapted with STDP, we next asked what sort of commensurate changes occur in the structural network to facilitate the observed synchronization. We expected that developing synchronous activity would necessitate strong intercluster connections. It is well-known that the STDP model implemented in our networks will lead to a bimodal synaptic weight distribution (Song et al., 2000), and we also saw a similar result in our stabilized networks (Figures 3A,B). From this distribution, we defined high strength connections as strengths >50% of the maximum (normalized strength > 0.5) and saw that a significantly higher fraction of intercluster connections were high strength than upstream intracluster outputs (intercluster: 0.822 ± 0.007 vs. intracluster: 0.521 ± 0.001; paired Student’s *t*-test, *p* < 10^–5^). In addition, the proportion of high strength intercluster connections increased rapidly and persisted for the duration of the simulation (Figure 3C). This remained true whether the network displayed high, moderate, or low levels of synchronization. Not only was the proportion of high strength intercluster connections stable, these connections themselves were highly stable. Among them, only 0.08 ± 0.04% change per minute was observed in the last 30 min of simulation time. As more intercluster connections were added (i.e. the proportion of excitatory inputs to downstream Cluster 2 from upstream Cluster 1 increased), the proportion of high strength intercluster connections decreased (linear regression, *R*^2^ = 0.58, *p* < 10^–5^) (Figure 3D). At low synchronization, when there were few intercluster connections, a larger proportion of those connections were high strength. As more intercluster connections were added, synchronization increased (Figure 2C), and the proportion of high strength intercluster connections decreased (linear regression, *R*^2^ = 0.58, *p* < 10^–5^) (Figure 3D). This suggests redundancy at maximal levels of coupling since it is unnecessary for as many connections to have high strength to achieve high synchronization.

Given the high strength intercluster connections, we considered whether intercluster projecting neurons (Inter Pre) are strong overall. To determine whether that was true, we assessed the average output strength of each excitatory population. The output strength of each neuron was summed and normalized by the total number of outputs. Contrary to our expectation, the Inter Pre population did not have high strength outputs as a whole, indicating that the outputs of these neurons to other neurons within the upstream population are rather weak. Instead, downstream neurons receiving connections from the upstream cluster (Inter Post) had significantly higher average output strength than other populations did (one-way ANOVA, *p* < 10^–5^) (Figure 3F). Interestingly, upstream neurons with no downstream projections (Intra Pre) showed significantly lower average output strength than did the intercluster populations (one-way ANOVA, *p* < 10^–4^) (Figure 3F). Notably, the Intra Pre neurons also had the least variance in strength, which suggests they respond minimally to the addition of intercluster connections (Figure 3F). Since the Intra Pre neurons also display relatively weak outputs, these findings show that Intra Pre neurons are the most isolated subpopulation and likely function primarily as drivers of activity in the upstream cluster.

### Controllability

At this point, we knew that the network synchronized and adapted structurally. However, we did not know how this architecture might be described with higher level network metrics, and specifically, whether the neuron subtypes we defined could be identified with these metrics. In network science, there are many measures that characterize nodal importance and identify nodes as influential under different circumstances. One such nodal property, betweenness centrality, describes how often paths between two nodes in the network must pass through a given node. High betweenness centrality indicates that node is an important connector between other nodes. Commonly called hubs, nodes with high betweenness centrality are often affected after TBI due to axonal injury (Fagerholm et al., 2015). A second nodal property, controllability, predicts the importance of nodes for driving the network to a different energetic state. We examined two mechanisms of control – average and modal, which describe the ability to access easy-to-reach and difficult-to-reach states, respectively. We were interested in how the network control points identified by average and modal controllability reflected the known dynamics of the system, namely synchronization. From all tested combinations of a 6 Hz Cluster 1 projecting to 4 Hz Cluster 2 (Figure 2C), two representative networks (one each for moderate and high synchronization) were selected for this analysis, though similar results were found for a more extensive sample of networks. Low correlation networks were also considered but were structurally similar to moderate correlation networks in this analysis.

**FIGURE 4:**
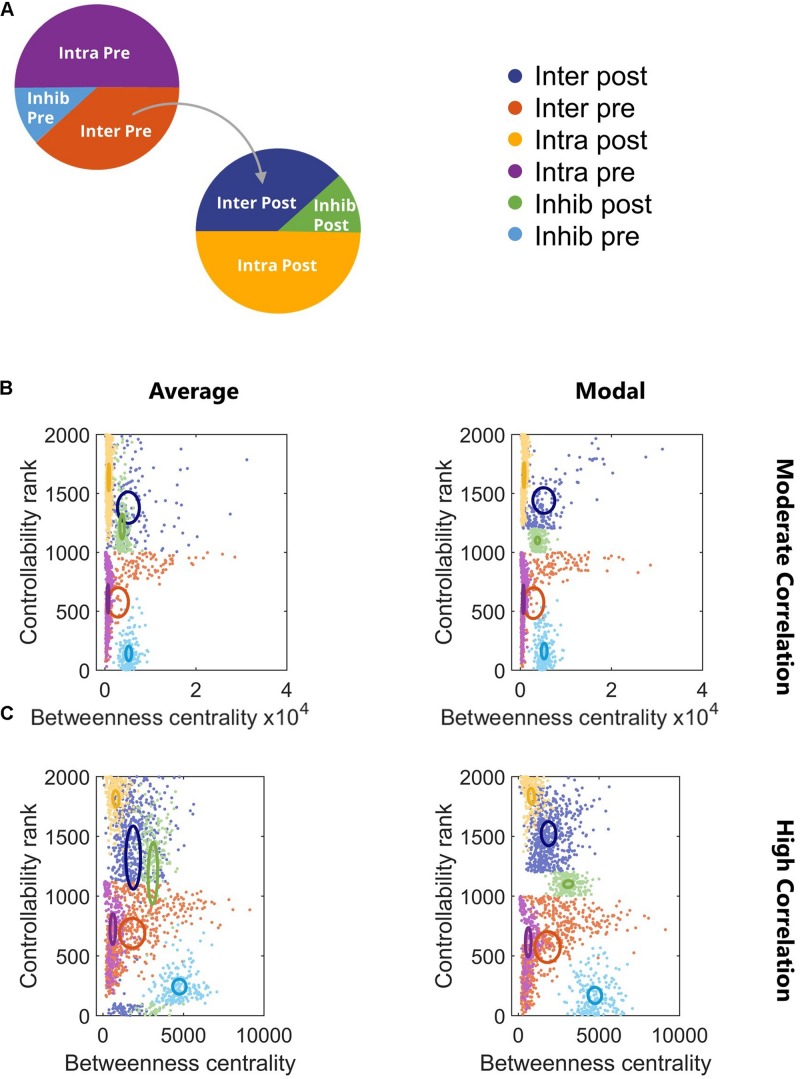
Controllability and betweenness centrality reveal phenotypes of neuron subtypes. **(A)** Legend for subsequent panels. **(B)** Controllability and betweenness centrality differentiated the neuronal subtypes for all correlation levels. All subtype comparisons were significant (repeated measures model with Tukey–Kramer *post hoc*, *p* < 10^–4^) with the exception of Inhib Post vs. Inhib Pre (*p* = 0.075). **(C)** All subtype comparisons were significant (repeated measures model with Tukey–Kramer *post hoc*, *p* < 10^–4^) with the exception of Inter Pre vs. Intra Post. Ovals are centered at the group mean and represent 50% of the group standard deviation.

We found that controllability and betweenness centrality reveal distinct phenotypes in this two-cluster architecture that mirror the subtypes we know to exist and previously defined (Figure 4). The subtypes with intercluster connections (Inter Pre and Inter Post) had the highest betweenness centrality, underscoring their integral position in the network. Any signal passing from Cluster 1 to Cluster 2 must pass through Inter Pre and Inter Post neurons. The betweenness centrality of these populations decreased as more intercluster connections were added and the correlation of the network increased (Figures 4B,C). In contrast to betweenness centrality, controllability did not show a relationship with correlation (Figures 4B,C). In general, populations in the downstream cluster had higher controllability than populations in the upstream cluster. This result indicates that targeting the downstream cluster would be a more effective way to change the network state than targeting the upstream cluster. For the hypothetical example of attempting to change the network state by breaking synchronization, exogenous stimulation applied to the downstream cluster would likely be a more effective strategy because the upstream cluster is the driver while the downstream cluster is the follower. Controllability does depend on the strength of connections, so while this was generally the case, we did identify a network in which the upstream cluster had higher controllability (data not shown). Overall, the subtypes showed minimal overlap in controllability, which emphasizes the distinct roles neuronal subtypes play in this two-cluster topology. Notably, average and modal controllability show similar trends, suggesting that the same populations would be important for driving the network to both easy-to-reach and difficult-to-reach states. Using a repeated measures model with Tukey–Kramer *post hoc* test for multiple comparisons, we found for high correlation networks all comparisons were significant (repeated measures model with Tukey–Kramer *post hoc*, *p* < 10^–4^) except for Inter Pre vs. Intra Post (Figure 4C). For moderate correlation networks, all subtypes were significantly different (repeated measures model with Tukey–Kramer *post hoc*, *p* < 10^–4^) with the exception of Inhib Post vs. Inhib Pre (*p* = 0.075).

### Injuring Highly Controllable Neurons by Subtype

Given the emergence of nodal subtypes, we sought to better understand their functional roles by implementing a scheme of targeted neurodegeneration in which we removed neurons from the network. Neurons were selected from one subtype at a time to compare the effect of their removal on synchronization and activity oscillations. Since controllability is believed to link structure and function, enhancing the likelihood of activity changes due to damage, we interrogated the functional influence of removing highly controllable neurons. This is in contrast to previous work in which highly controllable neurons are stimulated (Betzel et al., 2016; Muldoon et al., 2016; Gu et al., 2017; Kim et al., 2018). We hypothesized that removing the most controllable neurons within a given subtype would be more detrimental to network function than removing random neurons from that subtype. The distributions of output weights from removed neurons vs. remaining neurons of the same subtype remain bimodal; however, for some cases of controllability-based removal, the removed neurons have many connections of relatively low output strength (Supplementary Figure S5). The representative high and moderate correlation networks used for our controllability analysis were also used in these studies (*N* = 5 networks per type). Low correlation networks were excluded because the baseline synchronization level could not drop further as a result of injury. We tested three injury levels (25, 50, and 75% removal) for each excitatory subtype (Inter Post, Inter Pre, Intra Post, and Intra Pre). While inhibitory neurons influence local spike timing and may thereby modulate synchronization indirectly, excitatory neurons directly affect synchronization and adapt according to STDP in our model. Thus, we focused our injury on excitatory subtypes. Finally, we found that the intercluster connection weights continued to follow the distributions shown in Figures 3A,B with predominantly strong connections (Supplementary Figure S6). Therefore, our subsequent analysis emphasizes the effects of injury on network activity.

**FIGURE 5:**
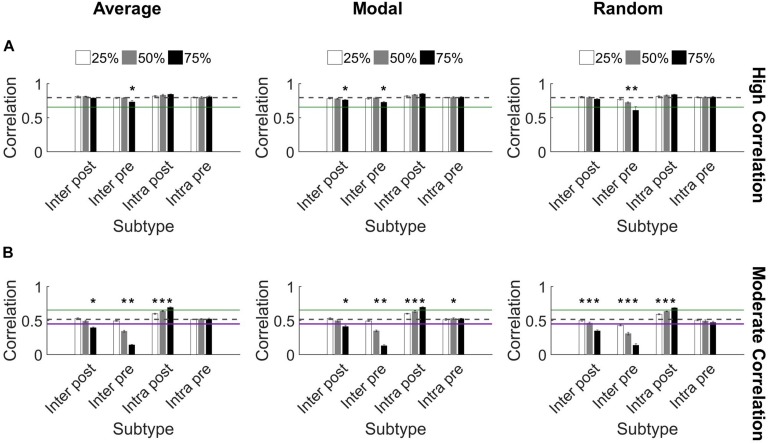
Synchronization protects against damage to microcircuits. **(A)** Most highly synchronized networks (correlation > 0.65; green line marks threshold for high correlation) maintained high correlation when neurons from specific populations were deleted from the network. The dashed gray line denotes the baseline correlation prior to injury. Some injured networks remained high correlation while having significantly lower synchronization compared to baseline (one-sided paired *t*-test, Bonferroni corrected, *p* < 0.0014). **(B)** In comparison, networks with moderate correlation (0.45 < correlation < 0.65) prior to injury were more likely to change synchronization level after injury. The most harmful deletion strategy was targeting excitatory neurons from Cluster 1 that send projections to Cluster 2 (the Inter Pre subtype). The green line marks the threshold between moderate and high correlation networks (0.65). The purple line marks the threshold between low and moderate correlation networks (0.45) (Figure 2). The gray dashed line marks the baseline correlation prior to injury. Many injury networks had significantly higher or lower correlation compared to baseline (paired *t*-test, Bonferroni corrected, *p* < 0.0014). Damaging Inter Pre neurons decreased synchronization while damaging Intra Post neurons increased synchronization.

We found that synchronization in high correlation networks was robust. When neurons were targeted according to their *controllability* ranking (average or modal), no level of deletion for any subtype reduced synchronization below the threshold for high synchronization (0.65 as determined in Figure 2C) (Figure 5A). We used paired *t*-tests with Bonferroni correction for multiple comparisons to evaluate each set of damaged networks compared to baseline uninjured networks. While there were a few significant decreases in synchronization (75% injury to Inter Pre neurons differed significantly from baseline for all targeting methods; *p* < 0.0014 for all), high correlation networks remained high correlation networks post-injury, with a single exception (Figure 5A). The one exception is *random* targeting of upstream neurons with intercluster connections (Inter Pre) at the highest injury level: 75% deletion yielded 0.6 ± 0.05 correlation. By applying ANCOVA to control for the injury level covariate, we also found that networks with damaged Inter Pre populations differed from one another based on the targeting strategy. Average and modal controllability targeting methods both differed from random deletion (ANCOVA with Bonferroni correction, *p* < 0.001); however, they did not differ from one another (*p* > 0.8). Lastly, we tested correlated activity within each cluster and found intracluster correlations remain high after injury (Cluster 1: 0.97 ± 0.04 and Cluster 2: 0.86 ± 0.07).

In contrast, the moderate correlation networks revealed a marked, dose-dependent vulnerability when the Inter Pre subtype (upstream neurons that send intercluster projections) was damaged (Figure 5B). While the changes were more modest than for Inter Pre, targeting the Inter Post population (downstream neurons that receive intercluster projections) also produced a dose-dependent decrease in synchronization. When comparing the results of Inter Pre deletion across the three methods, average and modal controllability-based deletion differed significantly from random (ANCOVA with Bonferroni correction, *p* < 0.008) but not from each other (*p* > 0.8). As for high correlation networks, intracluster correlated activity remained high (Cluster 1: 0.97 ± 0.03 and Cluster 2: 0.83 ± 0.06). For moderate correlation networks, we observed both significant decreases and increases in synchronization compared to baseline depending on the targeted subtype (paired *t*-test with Bonferroni correction, *p* < 0.0014) (Figure 5B). Notably, when Intra Post neurons were targeted, the resulting correlation increased. This is likely because achieving high synchronization is easier when there are fewer downstream neurons without direct inputs from the upstream cluster. In total, these results reveal a malleability of the synchronization of moderate correlation networks. Targeted injury could drive the network toward a state of either higher or lower synchrony.

**FIGURE 6:**
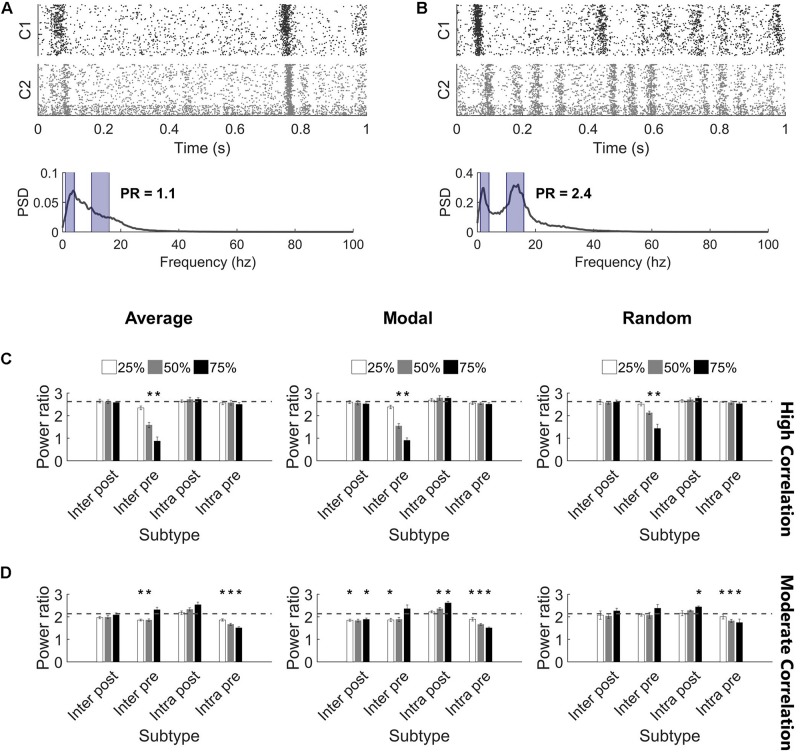
Highly synchronized networks are prone to large decreases in power ratio after injury. **(A)** A raster plot and corresponding frequency spectrum of an injured network with *high correlation* and *low power ratio*. The blue overlays mark the portions of signal that contribute to the power ratio calculation. **(B)** A raster plot and corresponding frequency spectrum of an injured network with *low correlation* and *high power ratio*. The blue overlays mark the portions of signal that contribute to the power ratio calculation. **(C)** Removing Inter Pre neurons in a high correlation network reduced the power ratio at deletion levels 50% and above for all selection methods (paired *t*-test, Bonferroni corrected, *p* < 0.0014). The dashed gray line marks the baseline power ratio prior to injury. **(D)** Removing neurons in a moderately correlated network had variable effects. In most cases, networks had modest, though significant, reductions in the power ratio; however, there were also injured networks with higher power ratio than they had at baseline (paired *t*-test, Bonferroni corrected, *p* < 0.0014). Increased power ratio was typically observed after damage to Intra Post neurons whereas decreased power ratio was common after damage to other subtypes.

While injury predominantly did not impact the synchronization of high correlation networks, we observed that the oscillation pattern of the high activity periods changed (Figures 6A,B). Therefore, we turned to the frequency spectrum to evaluate these rhythms. In undamaged networks, we routinely observed two prominent peaks in the power spectrum, corresponding to two primary oscillation frequencies that existed in the network activity (10–17 and 1–4 Hz; see the section “Materials and Methods,” Figures 2A,B, and Supplementary Figure S3 for further detail). The baseline power ratio between these two frequency bands (power in 10-14 Hz over power in 1-4 Hz) in high correlation networks was 2.6 ± 0.1 (*N* = 5). High correlation networks showed a rapid decline in this power ratio following selective damage to the Inter Pre population (paired *t*-tests with Bonferroni correction, *p* < 0.0014 for 50 and 75% injury for all selection strategies) (Figure 6C). A decrease in power ratio indicates a reduction in high frequency components of the activity signal. As we observed for correlation post-injury, average and modal controllability-based deletion differed from random deletion of the Inter Pre subtype (ANCOVA with Bonferroni correction, *p* < 0.005) but did not differ from one another (*p* > 0.8). Of note, this decrease in high frequency signal occurs across both clusters (Figure 6A) and suggests that the upstream cluster is unable to generate higher frequencies. Since the upstream cluster serves as the driver for high correlation networks, the downstream cluster depends on receiving input from the upstream cluster. After adapting with STDP, these networks appear to prioritize synchronization over more varied frequency information.

The power ratio of moderate correlation networks varied after targeted neurodegeneration. The baseline power ratio for moderate correlation networks was 2.1 ± 0.1 (*N* = 5). Removing non-projecting neurons from the upstream cluster (Intra Pre) significantly reduced the power ratio for all targeting methods (paired *t*-tests with Bonferroni correction, *p* < 0.0014) (Figure 6D). This effect was more pronounced in response to controllability-based deletion. In contrast, removing neurons in the downstream cluster that lacked intercluster connections (Intra Post) increased the power ratio (significant at the 75% level with random or modal controllability-based removal; paired *t*-tests with Bonferroni correction, *p* < 0.0014) (Figure 6D). The power ratio was most resilient to damage in the downstream population with intercluster input (Inter Post). Of note, the power ratio increased when the Inter Pre subtype was injured at the 75% level despite these same networks showing a decrease in correlation (Figure 5B). Here, the frequency of high oscillation periods in Cluster 1 decreased while Cluster 2 retained higher frequency (Figure 6B). Thus, for the aggregate network activity, frequency was high while correlation was low. In this case, the results of removing Inter Pre neurons were not significantly different by targeting method.

## Discussion

**FIGURE 7:**
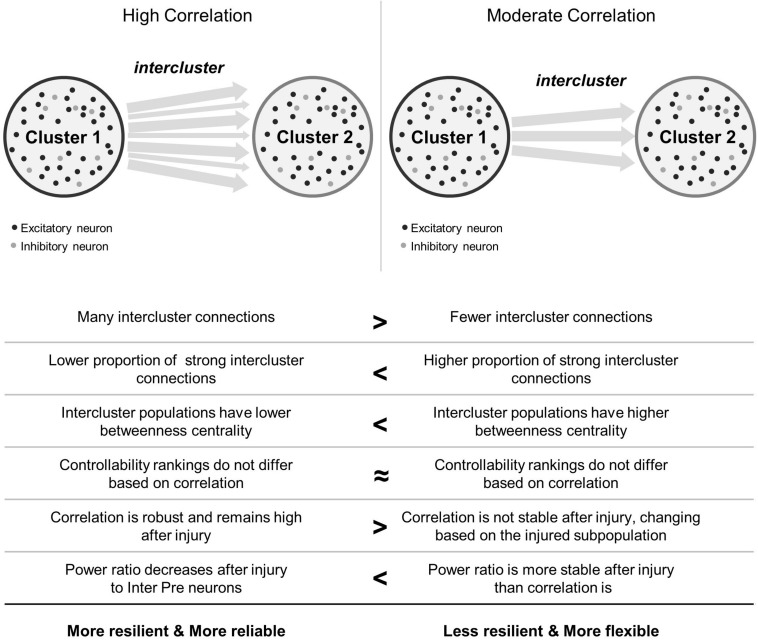
Summary comparison between high and moderate correlation networks. In the schematics showing network topology, black circles represent excitatory neurons and light gray circles represent inhibitory neurons. Not shown are the connections between them. Arrows between clusters stand for intercluster connections between excitatory neurons. Thicker arrows indicate stronger connections.

In this work, we were interested in how the coherence of two model microcircuits was established by connecting one population to another. We were also interested in determining whether specific neuronal subpopulations would be more influential in changing the dynamics of these coupled circuits after traumatic injury. We found that the two clusters synchronized with relatively few intercluster connections. In addition, intercluster connections became significantly stronger than did those among neurons within each microcircuit, indicating that they are high priority connections within the network. Finally, we employed targeted neurodegeneration to explore the influence of neuron subtypes on overall network behavior and showed that neuron controllability did not strongly influence injury response. However, neurons linking the two microcircuits were critical for maintaining both the broad power spectrum of activity communicated between the two networks and the coherence of this communication. Together, the results of targeted neurodegeneration reveal that densely connected microcircuits are resilient and highly reliable, even when injured, but these benefits may come at the cost of reduced signal flexibility (Figure 7). Conversely, moderately coupled microcircuits are more flexible than their densely coupled counterparts. However, because these networks have fewer intercluster connections, they are less resilient and may suffer greater effects of isolation after damage (Figure 7).

There are several assumptions we made throughout these studies. First, we used generic excitatory and inhibitory neurons based on the Izhikevich integrate-and-fire neuron model (Izhikevich, 2003). These model neurons are simplistic but versatile, well-verified, and adequate for our purposes. Several past studies employed these models to study polychronous neural computation (Izhikevich, 2006), autaptic neuronal connections (Wiles et al., 2017), and dopaminergic modulation of brain oscillations (Kobayashi et al., 2017). Second, we implemented only AMPA and GABA receptor currents as well as one type of plasticity (STDP). Although adding additional receptors or dynamics could affect the precise timing of neuron activation, these changes would not likely impact our broad findings, which indicate synchronization is a robust phenomenon in a unidirectional architecture. These simplifications were also made deliberately to produce a realistic, yet efficient and tractable, neuronal network model. A third simplification we made was connecting the two clusters by unidirectional connections only. It is often assumed that brain regions are reciprocally connected in diffusion tractography or functional MRI (Buckner et al., 2009; Bullmore and Sporns, 2009; Damoiseaux and Greicius, 2009; Nakamura et al., 2009; Cabral et al., 2011; Rubinov and Sporns, 2011; Horn et al., 2014; Fagerholm et al., 2015). Our goal was to build a more principled view of how groups of neurons interact to produce a composite network signal. To do so required beginning with a simplified architecture. Moreover, this unidirectional architecture does appear in larger, network-based descriptions of the brain. For example, the hippocampus is predominantly unidirectionally connected (Hummos et al., 2014; Wheeler et al., 2015), and other structures like the hypothalamus have a combination of bidirectional and unidirectional pathways, including afferent inputs as part of the sensory circuitry and outputs to the brainstem (Lechan and Toni, 2000; Card and Swanson, 2013). Given these limitations, however, we plan to pursue more complex and anatomically accurate network topologies in future work. In particular, it would be interesting to combine more diverse and specific neuron types with known connectivity features of anatomical regions like the hippocampus.

In healthy brain networks, it is known that synchronization or coherence between distant brain regions is important for functions like attention, learning, and memory (Dü Zel et al., 2010; Clayton et al., 2015; Fries, 2015; Hanslmayr et al., 2016). Typically, coherence is discussed at the scale of whole brain imaging, such as fMRI BOLD, which has a temporal resolution on the order of seconds (Logothetis and Wandell, 2004). With this resolution, there are nuances of activity patterns which may not be observed, and synchronization remains important at intermediate spatial and temporal scales. Nonetheless, due to experimental constraints, early studies about local networks and neuron response focused on firing rate (Barlow, 1972; Newsome et al., 1989). Currently, with improved technology for measuring activity in multiple neurons or regions simultaneously (multielectrode arrays, *in vivo* calcium imaging), there is a growing emphasis on understanding the correlation of activity among neurons (Cohen and Kohn, 2011). There is an interest in what correlation might encode in comparison to firing rate alone and what it might mean at various timescales (Cohen and Kohn, 2011). It is valuable to consider how complex patterns may combine to generate the activity observed at larger spatial scales and longer time scales. This work aims to examine this phenomenon at an intermediate scale where subtle topology changes may impact synchronization.

Our general finding that clusters of neurons synchronize with a low proportion of intercluster connections finds support in the literature. For example, thalamic inputs are important drivers of activity in the primary visual cortex yet account for only 5% of synapses on cortical simple cells (Wang et al., 2010). The authors further suggest that spike synchrony may be a critical mechanism for ensuring reliable, efficient transmission when inputs comprise a small percentage of overall synaptic input. Within the context of TBI, it is well-known that diffuse axonal injury and white matter damage more broadly are associated with cognitive impairment (Sharp et al., 2011; Johnson et al., 2013; Fagerholm et al., 2015; Blennow et al., 2016). Our current work suggests that if two brain areas are connected with a high density of projections, a significant amount of axonal injury (disconnection) will be needed to disrupt synchronization between these areas. Conversely, our work also suggests a relatively rapid decline in synchronization if two brain areas are only weakly connected and the linking connections are damaged. By extension, our work predicts that TBI neurodegeneration is most problematic when it impacts long-range projections between brain regions, especially when these regions are not strongly connected. In addition to synchronization itself, our supporting result that intercluster connections become strong, stable connections corroborates evidence in the literature. It has been observed in dissociated cultures of hippocampal neurons that “loose synchrony” exists at weak connectivity (Penn et al., 2016). As connectivity strength increased, the mean phase shift between oscillations decreased as the network converged to a common oscillation frequency characterized by synchronous periodic bursts (Penn et al., 2016). More broadly interpreted, these changes in synaptic strength reinforce connections among brain areas and could protect against synchronization deficits that occur in disease or injury.

Our results studying the influence of neuron controllability on intercluster dynamics revealed a surprisingly consistent result – deleting nodes of either high average or high modal controllability achieved the same change in network dynamics. Controllability is frequently applied to undirected, symmetric networks at the full-brain scale (Gu et al., 2015, 2017; Betzel et al., 2016; Muldoon et al., 2016). In general, these past studies show that nodes with high average controllability drive the network to easy-to-reach energy states, whereas nodes with high modal controllability push the network into hard-to-reach states. In the brain, these types of controllability often pertain to different tasks and networks. For instance, high modal control is associated with cognitive control regions, and high average control is associated with the default mode network (Gu et al., 2015; Tang et al., 2017). Our results, though, predominantly showed no differential effect of deleting neurons with either high average or modal controllability. One possibility is that easy- and hard-to-reach states are near one another on the energy landscape, so this deletion process would produce indistinguishable results. However, our manipulation also fundamentally differs from previous control studies in macroscale brain networks because deleting neurons effectively subtracts energy from the system as evidenced by deficits in both firing rate (Gabrieli et al., 2019) and frequency power after injury. These changes indicate a global loss of energy after neurodegeneration. More often, controllability is used in the context of stimulation or adding energy to drive the network to a different energetic state (Betzel et al., 2016; Muldoon et al., 2016; Gu et al., 2017; Kim et al., 2018). Prior to neurodegeneration, our networks already exist in a stable energy basin, and subtracting energy by removing nodes does little to drive the network toward a different state. As such, it suggests that *a priori* controllability rankings may be limited in their ability to predict dynamic network changes from degenerating neurons.

Whereas controllability regulates network dynamics and state transitions, synchronization appears to operate ideally within a “sweet spot” regime. With excessive synchronization comes dysfunction, including seizures. Excessive synchronization also limits cognitive flexibility, an important component of switching between different task networks. Using blood flow to detect coordinated neural activity, fMRI determines which regions of the brain are functionally connected. Neurological diseases are known to impact functional connectivity, variably increasing or decreasing it. In general, hyperconnectivity is associated with cognitive dysfunction, including decreased cognitive flexibility (Mayer et al., 2011; Tang et al., 2011; Pang, 2015; Venkatesan et al., 2015), an attribute that enables the brain to attain and utilize diverse brain states (Tang et al., 2017). In contrast, hypoconnectivity is related to cognitive decline due to loss of neural resources, such as occurs in Alzheimer’s disease (Sheline and Raichle, 2013; Hillary et al., 2015). A reasonable expectation is that traumatic injury – either from degenerating neurons or from disrupted connections between them – will only decrease functional connectivity in the brain. However, functional connectivity can both increase and decrease after TBI (Bullmore and Sporns, 2009; Mayer et al., 2011; Pandit et al., 2013; Sharp et al., 2014; Venkatesan et al., 2015). Our work studying the degeneration of specific neurons within each population raises an intriguing new mechanism at the cellular scale that may help explain how TBI can promote either functional hyper- or hypoconnectivity. In our moderate networks subjected to neurodegeneration, we observed both increases and decreases in correlation depending on which neuron subtype was targeted. If injury affects predominantly neurons that send connections to other regions, we can expect coherence with those regions to decline and subsequent hypoconnectivity. We would expect a similar decrease in functional connectivity if the projections between two different brain areas declined, a potential effect of diffuse injury to the white matter tracts connecting these areas. However, if neurons with primarily local connections are damaged, the diversity of information in that region goes down and correlation increases, leading to hyperconnectivity and reduced cognitive flexibility. To our knowledge, we are not aware of previous work showing this bifurcating response within a single network, making this the first study to demonstrate both higher and lower synchronization as a result of differentially targeted injury.

Correlation, as we have defined it, is a robust metric with tight standard deviations and high consistency among simulations. Despite this, synchronization alone does not provide a full picture of network activity. The traditional metric of neuron firing rate also fails to add much to this picture because it does not account for the variability in action potential timing. Both our networks and more complex networks *in vitro* and *in vivo* develop oscillatory patterns with periods of high and low activity. These rhythms may themselves encode information or instead facilitate the flow of information (Sejnowski and Paulsen, 2006). *In vivo* oscillations contribute to many important cognitive functions, including the representation, consolidation, and retrieval processes of memory (Dü Zel et al., 2010; Hanslmayr et al., 2016). Oscillations are also believed to coordinate activity in different brain regions, dynamically shaping brain networks that have static structural connections (Dü Zel et al., 2010; Deco and Kringelbach, 2016). The coupling is hypothesized to occur via different frequencies. Theta-gamma coupling in the hippocampus is one well-studied example (Dü Zel et al., 2010; Lisman and Jensen, 2013; Colgin, 2015), in which gamma frequencies are coupled to phases of the theta signal to enable CA1 to coordinate with the entorhinal cortex via high frequency gamma and with CA3 via low frequency gamma (Dü Zel et al., 2010; Colgin, 2015). Similarly, coherent activity appears between the hippocampus and prefrontal cortex during certain behaviors in rodents (Jones and Wilson, 2005; Tamura et al., 2017). Thus, transmitting spike rate information across different frequency bands allows a single region to communicate with multiple regions or even participate in different networks simultaneously. As an approximation of the signal properties encoded in the network, we defined a power ratio of the total network activity. In a more complex topology, different features of the frequency spectra may synchronize more strongly than others between two regions. Our results indicate that weakly connected regions are more vulnerable to changes in synchronization post-injury while highly connected regions are more vulnerable to changes in frequency, though they may remain synchronized. As the brain is comprised of regions coupled by varied connectivity strength, our results imply that an injured brain may show altered synchrony or oscillation frequency between some brain regions and not others, with the difference due to the connection strength. Moreover, both phenomena may occur simultaneously for a given region, contributing to the response heterogeneity observed after TBI. We also note that the high frequency components were susceptible to neurodegeneration, showing the largest change when upstream projecting neurons were targeted in high correlation networks. This finding corroborates other reports of decreased broadband power in the CA1 region of the hippocampus (Paterno et al., 2016; Gagnon et al., 2019).

The changes in oscillatory rhythms in our model after damage lead us to consider ways to restore the original rhythms. One possibility is stimulation of neurons within each network, which would also enable us to further explore our insights about controllability in the framework of injury. At a larger scale, deep brain stimulation (DBS) has been implemented to treat neurological conditions including Parkinson’s disease (de Hemptinne et al., 2015) and chronic pain (Owen et al., 2006) by modulating inappropriate brain activity (Kringelbach et al., 2007). While it has been used for years, the fundamental mechanisms of DBS are not well understood. In the context of TBI, DBS has been previously proposed to restore cognitive rhythms (Pevzner et al., 2016). At the scale of our network model, we can examine the principles of restorative stimulation protocols as a means of reestablishing disrupted rhythms. With the flexibility of our model, we can compare various stimulation strategies, including testing different frequencies and targeting highly controllable neurons, to study both effectiveness and structural network changes. Past work indicates the controllability type and rank for a network node will affect transition states for the network when energy is injected into this node (Betzel et al., 2016; Muldoon et al., 2016; Kim et al., 2018). As such, we expect that nodal stimulation will function differently than nodal deletion and will allow one to systematically reconstruct activity oscillations and re-establish information encoding properties across nodes in the network.

In closing, we find that a relatively simple injury, namely neurodegeneration, can cause complex outcomes that depend on the baseline coupling of microcircuits and on the function of damaged neurons (Figure 7). The communication abilities (synchronization) and information coding capacity (frequency content) of these networks may be impaired after traumatic injury. Densely connected microcircuits possess an inherent resilience to synchronization-related changes after damage while moderately coupled networks are more malleable. Our work underscores that upstream neurons sending downstream projections are highly valuable for maintaining both synchronization and frequency properties of the aggregate signal in a multi-regional network. More broadly, this work raises a new dimension of heterogeneity of TBI where the pattern of cellular damage may contribute to the specific outcome and impairment. In future work, this complexity could be explored with a multiscale approach which integrates local, time-varying signal information as inputs to oscillator-based models of macroscale brain connectivity (Váša et al., 2015; Lee et al., 2017). Thus, this work facilitates integrative multiscale efforts for translating fundamental mechanisms of TBI to macroscale consequences by establishing principles which may be applied and tested in a larger scale model of the brain.

## Data Availability Statement

The datasets generated for this study are available on request to the corresponding author.

## Author Contributions

SS and DM conceived and designed the studies and analysis, and wrote the manuscript. SS performed the simulations and conducted the analysis. SS and DG contributed to the model and analysis tools. DG contributed to the manuscript discussion and revision.

## Conflict of Interest

The authors declare that the research was conducted in the absence of any commercial or financial relationships that could be construed as a potential conflict of interest.
