# Supplementary material for: Neuronal Degeneration Impairs Rhythms Between Connected Microcircuits
Source: Front Comput Neurosci. 2020 Mar 3;14:18. doi: 10.3389/fncom.2020.00018 (PMC7063469; doi:10.3389/fncom.2020.00018)
Supplement: Supplementary file 1 [file Data_Sheet_1.docx]

Supplementary Material

# Supplementary Figures


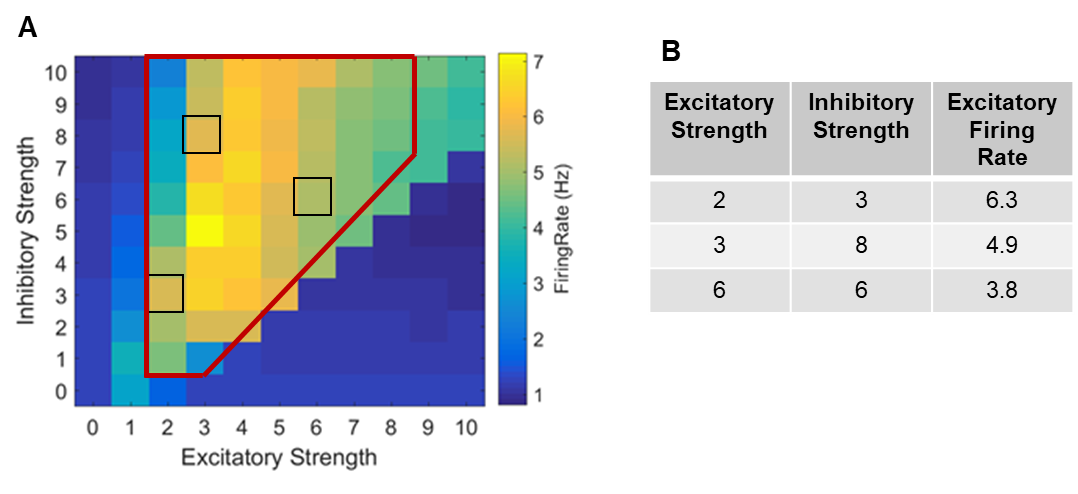


**FIGURE S1 | Preliminary testing to determine synaptic strength parameters. (A)** We tested all combinations of excitatory and inhibitory strength available in our model. This revealed different regimes of activity. Outside the region outlined in red, the activity with too low. (i.e. The activity was close to the noise floor of 1 Hz.) We chose examples with excitatory firing rates representative of approximately 4, 5, and 6 Hz (outlined by black squares). These values for strength are normalized. **(B)** The table shows the excitatory and inhibitory synaptic strengths used with the excitatory firing rate attained for each.

**
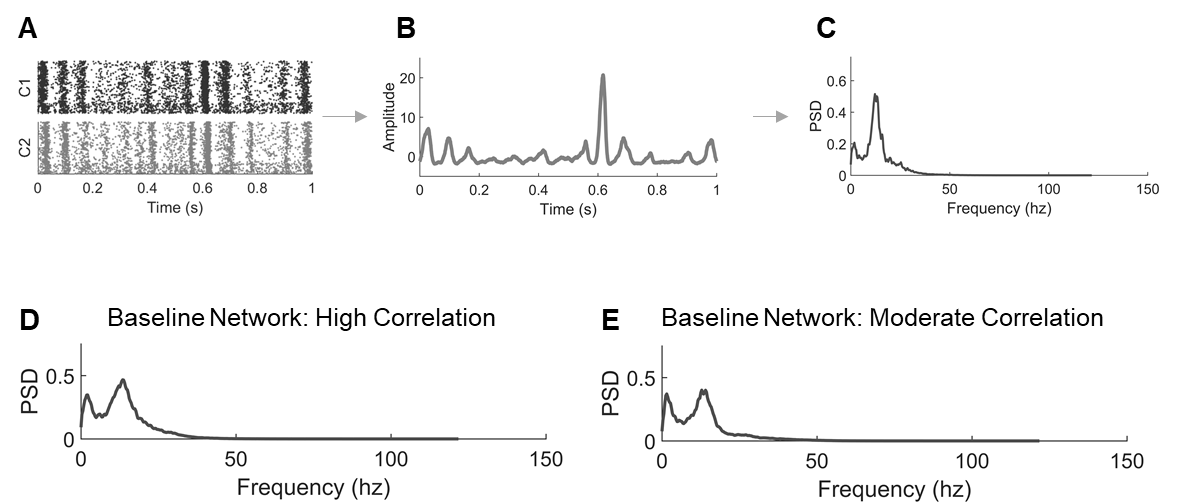
**

**FIGURE S2 | Frequency spectra for uninjured networks. (A)** A raster plot of raw activity in Cluster 1 and Cluster 2. **(B)** For rhythm analysis, the aggregate activity was summed across both clusters, and the signal was smoothed. **(C)** The aggregate signal was converted to the frequency spectrum by Welch’s method where PSD is the power spectral density. **(D)** Representative frequency spectrum for high correlation networks. **(E)** Representative frequency spectrum for moderate correlation networks.

**
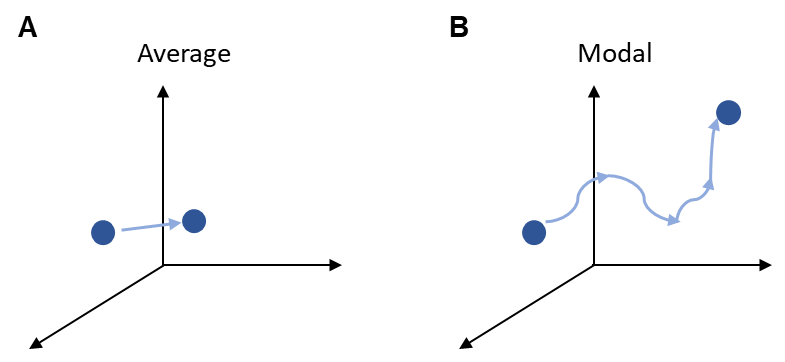
**

**FIGURE S3 | Controllability schematics. (A)** Average controllability refers to moving the network to a nearby, easy-to-reach state in the energy landscape. **(B)** In contrast, modal controllability refers to driving the network to a difficult-to-reach state with higher energy cost.


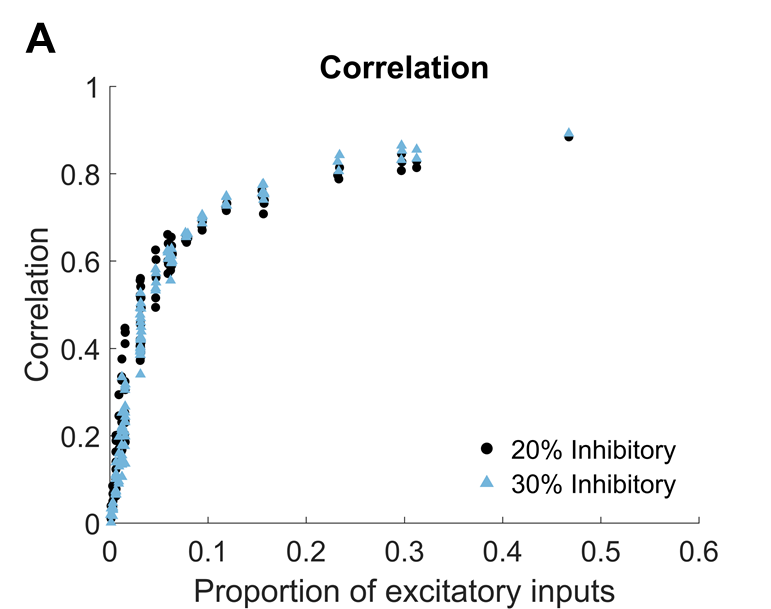


**FIGURE S4 | Synchronization with 30% inhibitory neurons. (A)** In a subset of simulations, we tested networks with 30% inhibitory and 70% excitatory neurons. In comparing to the 20% inhibitory and 80% excitatory ratio used in the rest of this work, we found that the synchronization behavior is substantially the same.

**
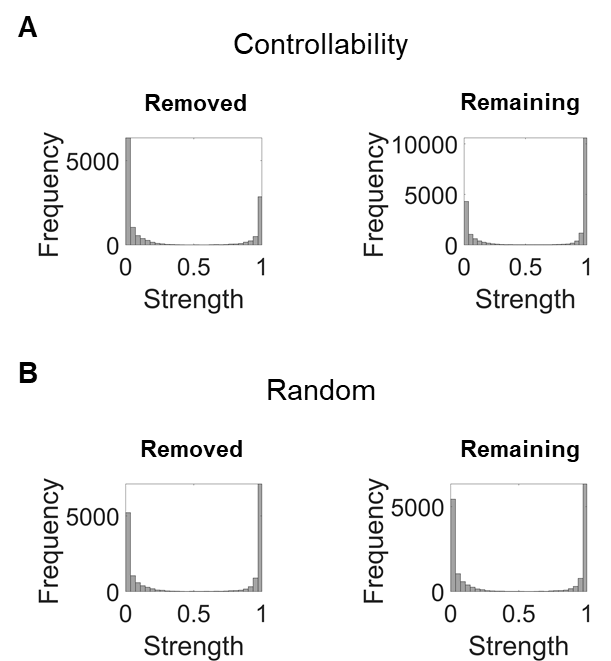
**

**FIGURE S5 | Connection weights of removed neurons.** These representative histograms are taken from cases of removing 50% Inter Pre neurons from a single moderate correlation network. The weights used are output weights from the beginning of the simulation. **(A)** The distribution of output weights of all removed neurons compared to remaining neurons of the same subtype at 50% removal for a representative controllability-based removal case. **(B)** The distribution of output weights of all removed neurons compared to remaining neurons of the same subtype at 50% removal for a representative random removal case.


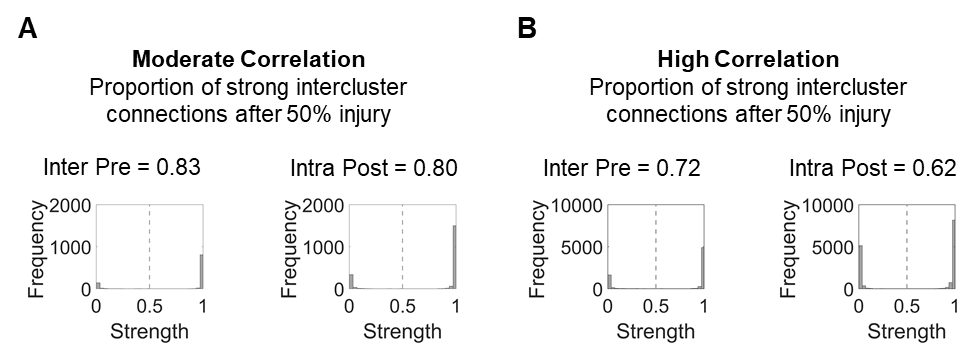


**FIGURE S6 | Intercluster connection weights after damage. (A)** For comparison with Figure 3, the histograms show the edge weights of intercluster connections after 50% random removal of neurons in different subtypes. For all moderate correlation networks, the proportion of strong intercluster connections after random injury is 0.80 ± 0.02. Two examples are shown for the removal of Inter Pre vs. Intra Post neurons. **(B)** For all high correlation networks, the proportion of strong intercluster connections after random injury is 0.64 ± 0.05. Two examples are shown for the removal of Inter Pre vs. Intra Post neurons.
